# BCL9/BCL9L in hepatocellular carcinoma: Will it or Wnt it be the next therapeutic target?

**DOI:** 10.1007/s12072-020-10059-5

**Published:** 2020-06-01

**Authors:** Akshata Moghe, Satdarshan P. Monga

**Affiliations:** 1 Division of Gastroenterology, Hepatology and Nutrition, Department of Medicine, University of Pittsburgh School of Medicine, Pittsburgh, PA USA;; 2 Pittsburgh Liver Research Center, University of Pittsburgh Medical Center and University of Pittsburgh School of Medicine, Pittsburgh, PA USA;; 3 Division of Experimental Pathology, Department of Pathology, University of Pittsburgh School of Medicine, Pittsburgh, PA USA

In the past few decades, hepatocellular carcinoma (HCC) has emerged as the most rapidly rising cause of cancer-related mortality in the United States [1]. Even though we are gradually getting a grip on the traditional risk factors of hepatitis C and B infections, the rising incidence of other risk factors such as alcohol-induced liver disease and non-alcoholic fatty liver disease (NAFLD) are posing continued challenges. Unfortunately, the 5-year survival rate remains dismal and is estimated to be only 18% [1]. This speaks to the lack of effective treatment options, and highlights the importance of rigorous research efforts to discover and develop novel therapeutic strategies. The only curative treatment options today are liver transplantation, surgical resection, and ablation; but these cannot be offered to the majority of patients with HCC, as most patients typically present with advanced disease that is not amenable to these treatment modalities. Systemic therapies such as sorafenib, regorafenib, lenvatinib, and nivolumab are used when curative options cannot be offered; but are only modestly efficacious and ridden with numerous side-effects. Hence, now more than ever, there is an acute need for efficacious therapies that focus on molecular and mechanistic targets in HCC.

We know that HCC is a heterogenous cancer, with each individual tumor arising from a distinct genetic and molecular signature. Treatment of HCC thus requires recognition and targeting of specific signaling pathways which are central to the process of carcinogenesis. It has been previously well-established that dysregulation of the Wnt/β-catenin signaling pathway plays a critical role in the development of HCC [2]. Amongst the multiple genetic and signaling alterations that can activate the Wnt signaling pathway, activating mutations in β-catenin gene *CTNNB1*, are the most common, ranging from 13–43% of all HCCs depending on the cohort being studied [3–5]. The β-catenin destruction complex is ineffective in phosphorylating and degrading β-catenin resulting in nuclear translocation of β-catenin [2]. In the nucleus, BCL9/BCL9L (B-cell lymphoma 9/B-cell lymphoma 9-like) and Pygopus act as transcriptional co-activators of β-catenin, and form a ‘Wnt enhanceosome’ along with T-cell factor-1/lymphoid enhancer factor-1 (TCF/LEF1) to drive the expression of downstream target genes. In light of this, Huge et. al. present their findings describing an oncogenic role for BCL9 and BCL9L in HCC, with relevance to the Wnt-activation status of the cancer [6].

In their paper, Huge et. al. first verify the relevance of BCL9 and BCL9L in human HCC by examining three separate publicly available HCC databases-TCGA-LIHC, GSE22058, and GSE25097, and demonstrating that BCL9 and BCL9L expression levels are significantly elevated in HCC tissue compared to adjacent non-HCC tissue in all three datasets. Previous studies have examined the role of BCL9 in cancer, including HCC [7–10]. Hyeon et. al. studied BCL9 in 288 primary HCC patients who underwent curative hepatectomy, and noted that ~25% of the HCCs showed nuclear BCL9 protein expression. BCL9 expression correlated with increased microvascular invasion, increased intrahepatic metastasis, and higher Edmondson grade. More importantly, BCL9 appeared to be a marker of shorter disease-free survival after curative hepatectomy, but this trend did not reach significance (p=0.078). Other studies have also shown similar findings, with inhibition of HCC migration, invasion and angiogenesis due to loss or downregulation in BCL9 [8, 10]. Huge’s group confirmed this finding to be true with regard to not only BCL9, but also BCL9L, and in fact, larger tumors had higher BCL9L expression compared to small tumors.

Interestingly, there is only a single study examining both BCL9 and BCL9L in mouse livers [9]. This recent study by Gay et.al. shows that BCL9/BCL9L play an instrumental role in the Wnt-β-catenin pathway driven hepatocyte proliferation as well as transformation. Using a hepatocyte -specific adeno-associated virus (AAV) - Cre recombinase system, the authors showed that deletion of BCL9 and BCL9L in aged mice resulted in a small but significant reduction in liver-to-body weight ratio compared to controls. There was also reduction in glutamine synthetase, a Wnt target gene and zonation marker, 140 days after induction. To investigate the role of BCL9/BCL9L in the Wnt-β-catenin pathway, the authors then expressed a single copy β-catenin mutation in the mice with and without BCL9/BCL9L deletion. Expression of mutant β-catenin caused upregulation of Wnt-β-catenin target genes and hepatomegaly with a liver failure phenotype in 2 weeks in the mice. Deletion of BCL9/BCL9L completely abrogated this phenotype and the mice aged up to 140 days, showing significantly prolonged survival. Examination of both the phenotypes at day 14 after induction showed that deletion of BCL9/BCL9L downregulated Wnt targets, reduced proliferation examined by BrdU studies and notably, also suppressed the glutamine synthetase positivity driven by mutant β-catenin [9]. They did not examine the effects of differentially deleting BCL9 and BCL9L, likely because they are thought to work in tandem and be largely redundant in their function. However, this assumption is now questioned by the work of Huge et. al. since they observed reduction of Wnt-β-catenin signaling with deletion of BCL9L, but not BCL9. The authors provide a thoughtful analysis of these findings, raising the question of possible one-way compensation for loss of BCL9, but not for loss of BCL9L. They also suggest differential expression of BLCL9 and BCL9L in different HCC cell lines as a possible explanation, and maybe BCL9L is the major protein in the cell lines examined [6]. It will be interesting to further examine the independent functions and interplay of BCL9 and BCL9L, both in HCC and other cancers in order to better characterize their relationship.

The most intriguing finding in the study by Huge et.al. is that they do not observe ‘Wnt-responsiveness’ of BCL9 and BCL9L. They claim this because Wnt-active HCC cell lines and primary HCCs neither showed higher expression of BCL9/9L, nor was BCL9/9L expression enhanced by induced Wnt activation. The authors suggest that this may be a cell-type specific phenomenon, as BCL9/9L have been shown to be Wnt-responsive in colon cancer [11]. However, even other liver-specific studies in ‘Wnt-active’ Huh6 and HepG2 HCC cell lines have noted increased Wnt signaling with BCL9 overexpression, contrary to the results of the authors. On the other hand, BCL9 suppression in Wnt-inactive HLE cells increased expression of target Wnt gene AXIN2 in their experiments. Even more interestingly, there seems to be a disconnect between the Wnt signaling and functional effects of BCL9 knockdown, as BCL9 knockdown did not affect cell survival in Wnt-active HCC, but decreased cell survival in Wnt-inactive HCC. This raises the question of whether the system is affected downstream of Wnt, and if β-catenin is activated without Wnt activation in Wnt-inactive cell lines. This would still not fully explain why Wnt-active cell lines are not affected by BCL9 knockdown. Certainly, β-catenin activation has been observed in the absence of *CTNNB1* mutations such as in HCC driven by TGFβ [12]. It will be important to further study the roles of BCL9/9L in HCCs with such non-classical activation of β-catenin.

The authors rightly note that although BCL9/9L are best known for their roles as co-activators of β-catenin, their findings suggest a Wnt-independent role for these proteins. This has been explored and demonstrated in other cell lines such as ameloblasts and intestinal cells, but hitherto uncharted territory in the liver [13, 14]. BCL9/9L are certainly promising targets in the therapy of HCC, and small molecule inhibitors of BCL9/9L have already been developed[15–17]. However, critical questions regarding their independent and combinatorial roles(s), and interplay with other proteins need to be answered before BCL9/9L can take centerstage as the next big stars in the therapy of HCC.
